# *In vitro* and *in vivo* evaluation of alginate hydrogel-based wound dressing loaded with green chemistry cerium oxide nanoparticles

**DOI:** 10.3389/fchem.2023.1298808

**Published:** 2023-11-23

**Authors:** Ran Zhao, Chenyuyao Zhao, Yi Wan, Muhammad Majid, Syed Qamar Abbas, Yibing Wang

**Affiliations:** ^1^ Burn and Plastic Surgery, Shandong Provincial Hospital Affiliated to Shandong First Medical University, Jinan, Shandong, China; ^2^ Key Laboratory of Biopharmaceuticals, Postdoctoral Scientific Research Workstation, Shandong Academy of Pharmaceutical Science, Jinan, Shandong, China; ^3^ Graduate School, Shandong First Medical University, Jinan, Shandong, China; ^4^ School of Mechanical Engineering, Shandong University, Jinan, Shandong, China; ^5^ Faculty of Pharmacy, Hamdard University, Islamabad, Pakistan; ^6^ Department of Pharmacy, Sarhad University of Science and Technology, Peshawar, Pakistan

**Keywords:** wound healing, alginate, cerium oxide nanoparticles, curcumin, hydrogel

## Abstract

Interactive wound dressings have displayed promising outcomes in enhancing the wound healing process. This study focuses on creating a nanocomposite wound dressing with interactive and bioactive properties, showcasing potent antioxidant effects. To achieve this, we developed cerium oxide nanoparticles utilizing curcumin as both the reducing and capping agent. Characterization techniques such as SEM, EDX, DLS, Zetasizer, FTIR, and XRD were utilized to analyze the cerium oxide nanoparticles synthesized through a green approach. The image analysis on the obtained TEM images showed that the curcumin-assisted biosynthesized CeO_2_NPs have a size of 18.8 ± 4.1 nm. The peaks located at 28.1, 32.7, 47.1, 56.0, 58.7, 69.0, and 76.4 correspond to (111), (200), (220), (311), (222), (400), and (331) crystallographic planes. We applied the Debye–Scherrer equation and observed that the approximate crystallite size of the biosynthesized NPs is around 8.2 nm based on the most intensive broad Bragg peak at 28.1°. The cerium oxide nanoparticles synthesized were integrated into an alginate hydrogel matrix, and the microstructure, porosity, and swelling behavior of the resulting wound dressing were assessed. The characterization analyses provided insights into the physical and chemical properties of the green-synthesized cerium oxide nanoparticles and the alginate hydrogel-based wound dressing. *In vitro* studies demonstrated that the wound dressing based on alginate hydrogel exhibited favorable antioxidant properties and displayed hemocompatibility and biocompatibility. Animal studies conducted on a rat full-thickness skin wound model showed that the alginate hydrogel-based wound dressing effectively accelerated the wound healing process. Overall, these findings suggest that the alginate hydrogel-based wound dressing holds promise as a highly effective material for wound healing applications.

## 1 Introduction

With advancements in nanotechnology and nanoscience, novel nanomaterials have surfaced, raising concerns about their potential impact on human health and the environment. However, amidst these concerns, a group of scientists is actively refining eco-friendly techniques for manufacturing metal and metal oxide nanoparticles (Hutchison, 2016). It is preferable to use green nanotechnology in production and application since it reduces dangers related to nanotechnology (Hutchison, 2008; Ali et al., 2022; Talha et al., 2022). The emergence of engineered nanoparticles is a significant milestone in the fields of nanotechnology and materials engineering. Nanotechnology has revolutionized the pharmaceutical industry and various areas of medicine, leading to numerous advancements. However, it is crucial to transition these innovations from the confines of the laboratory to practical applications in the real world in order to fully harness their potential. Engineered nanoparticles have become prevalent across various industries, with their presence particularly noticeable in sectors such as personal care, cosmetics, and clothing (Hobson, 2009; Shi et al., 2010; Mahmoodi et al., 2023).

In order to drive human progress and global development, the commercialization of disruptive technologies plays a pivotal role. However, it is imperative that we approach this process with caution, considering the capabilities, health implications, and environmental impact of these innovations. One such technology that requires immediate attention is nanoparticles (NPs), as they pose significant risks to human health. Unfortunately, their manufacturing and distribution often occur without proper regulation, particularly in the expanding global market.

While cutting-edge chemical processes are being developed with a strong emphasis on safety, it is crucial to adhere to a set of universal principles that aim to reduce or eliminate potentially harmful substances. This alignment with such principles is an integral aspect of the burgeoning field of green chemistry (Xia et al., 2009; Boregowda et al., 2021; Kumari et al., 2021; Kashyap et al., 2023).

The skin, the largest organ in the human body, covers approximately 2 square meters. It serves as a vital protective barrier against the harsh external environment. In addition to its role as a shield, the skin performs several essential functions. It helps maintain moisture levels, enhances sensory perception, regulates body temperature, promotes the equilibrium of bodily fluids, and acts as a defense against foreign infections (Peate, 2021; Alalaiwe et al., 2023; Panieri et al., 2023). The skin possesses impressive adaptability, allowing it to withstand a range of environmental stressors over time. However, when the skin sustains damage, it can give rise to potential health complications. Emergency rooms frequently encounter various types of injuries, including burns, surgical incisions, contusions, scrapes, and trauma-induced scratches. These injuries require prompt medical attention and care (Janda et al., 1997; Hassan et al., 2022; Mlambo et al., 2022; Kumari et al., 2023). The wounds of most living things heal within 3 months, depending on their severity, thanks to their self-healing abilities. The process of wound healing is becoming increasingly challenging for clinicians, making any new materials or methods highly sought after. Massive advancements in nanotechnology, especially in nanochemistry and nanomanufacturing, have completely altered the pharmaceutical and biotechnology sectors. Because of their unique structure, nanomaterials (those with at least one dimension below 100 nm) exhibit unusual physical and chemical characteristics, such as enhanced quantum tunneling at small and large scales. Due to their increased adsorption capacity, antibacterial characteristics, and medication loading, nanomaterials have also recently seen widespread application in wound healing (Scrinis and Lyons, 2007; Pachuau, 2015; Huang et al., 2021; Malik et al., 2023; Zhao et al., 2023).

For its controlled synthesis and catalytic uses, CeO_2_ has been widely researched due to its high oxygen vacancy content and reversible transformation between Ce (iii) and Ce (IV). It has come to light in recent years that CeO_2_ nanoparticles can function as free radical scavengers, neutralizing harmful reactive oxygen and nitrogen species (ROS and RNS) like superoxide radicals, hydrogen peroxides, hydroxyl radicals, and nitric oxide radicals (Hojo et al., 2010; Rong et al., 2020). This has sparked growing interest in their possible biomedical applications. CeO_2_ nanoparticles have shown promise in preventing a variety of diseases linked to oxidative stress, including chronic inflammation, ischemic stroke, and neurological disorders, according to preliminary biological research. It has been found that CeO_2_ nanoparticles promote the growth and migration of major skin-producing cells, which in turn speeds up the healing of cutaneous wounds (Celardo et al., 2011; Wason and Zhao, 2013; Siposova et al., 2019; Allu et al., 2023). However, the impact of CeO2 nanoparticles on molecular biology during the wound healing process has not been published as of yet, to the best of our knowledge.

Lately, there has been increasing interest in studying the antioxidant potential of cerium oxide nanoparticles (nanoceria). It has been suggested that nanoceria could potentially scavenge superoxide radicals, hydrogen peroxide radicals, hydroxyl radicals, and nitric oxide radicals (Ganesana et al., 2012; Yadav et al., 2019; Zhao et al., 2023). Therefore, nanoceria has garnered significant attention in biological research due to its potential ability to prevent tissue damage caused by radiation, shield the retina from laser light, extend the lifespan of photoreceptor cells, reduce the severity of spinal cord injuries, alleviate chronic inflammation, and enhance angiogenic processes (Gao et al., 2014; Andrabi et al., 2020). Considering the advantageous characteristics of CeO_2_ nanoparticles (NPs) and the utilization of a sustainable green synthesis approach, our team has opted to create innovative bioactive CeO_2_ NPs by incorporating a potent natural substance, curcumin. We have then employed these CeO_2_ NPs as a bioactive component within interactive wound healing materials, maximizing their therapeutic properties. According to a report, the amalgamation of cerium oxide nanoparticles and curcumin has been found to enhance the stability of curcumin, hence, potentially offering therapeutic benefits for a certain disease. Several studies have documented the potential benefits of an effective cerium oxide-curcumin combination in terms of enhanced bioavailability and decreased toxicity, hence, aiding in the treatment of chronic illnesses (Andrabi et al., 2020). CeO_2_ nanoparticles (NPs) were synthesized and then integrated into alginate hydrogel for their application as wound healing materials. Characterization techniques and biological evaluations demonstrated that these developed wound healing materials are interactive, bioactive, and hold great potential, as they displayed significant biological activities.

## 2 Materials and methods

### 2.1 Materials

Curcumin, dimethyl sulfoxide (DMSO), K_2_CO_3_, CaCl_2_, Cerium nitrate (Ce (NO_3_)_3_·6H_2_O) with 99.9% purity (trace metal basis), and Alginate were obtained from Sigma-Aldrich as a reagent-grade chemical. DMEM cell culture medium, FBS, and antibiotics (Pen/Strep) were obtained from Invitrogen.

### 2.2 Instrumentation

The FTIR spectrum of samples was obtained using Shimadzu 8101M FTIR (Kyoto, Japan) at ambient conditions. The samples were mixed with potassium bromide (KBr) and pressed to form a pellet. SEM (Jeol JSM-6390 SEM) and EDX detector (Oxford Link SATW ultrathin window) equipped SEM were applied to obtain SEM micrographs and semi-quantitative elemental analysis. The samples were coated with conductive elements to avoid any interferences with the imaging and analyzing process. A TEM microscopy (Zeiss Leo q06) was used to provide proper TEM images from the NPs at 200 kV accelerating voltage. The NPs were dispersed in solution with low concertation and a small amount of the dispersed NPs (10 µL) was poured on the TEM grid and allowed to dry. A Zetasizer Nano ZS90 was applied to measure the hydrodynamic size and zeta potential of NPs at RT. The UV-vis spectrum of the synthesized NPs was recorded using a spectrophotometer (Shimadzu 1650 PC UV-vis spectrophotometer. Kyoto, Japan). Siemens D5000 diffractometer (Aubrey, Texas, United States) was applied to record the X-ray diffraction (XRD) pattern of the synthesized NPs.

### 2.3 Synthesis of NPs

The green synthesis method was applied to obtain CeO_2_ NPs. In this approach, the applied natural/herbal substance (curcumin in this study) is applied as the reducing agent and stabilizing/capping agent for the supplier source of cerium (cerium nitrate salt in this study). First, Cur was dissolved in DMSO with the final concentration of 20 mM, then 120 µL of the prepared Cur/DMSO solution was diluted with 7 mL of DI water. Next, K_2_CO_3_ (150 mM) was used (drop-wise) to adjust the pH of the solution in 9–10. The as-prepared solution was stirred for 10 min to induce the release of hydrogens from hydroxyl groups of Cur (a critical step that makes Cur a reducing agent). Then, the cerium nitrate solution (1 mM) was added drop-wise to the Cur solution and allowed to stir until the formation of a white precipitate. After 2 days, the reaction was complete in this colloidal solution. Smaller particles formed as the concentration of Cur increased. Using centrifuge filter tubes (Amicon Ultra-50 C) and heating them to 4,000 rpm for 4 min at room temperature, we were able to remove all unreacted Cur and salt from our samples. Four rounds of centrifugation were performed until no UV-Vis absorbance spectra showed evidence of Cur.

### 2.4 Characterization of NPs

The synthesized Cur-CeO NPs were characterized with different and related techniques. The synthesized Cur-CeO NPs were dispersed in DI water and sonicated thoroughly and their UV-vis spectrum was recorded. Moreover, the UV-vis spectrum of free Cur was recorded. The functional groups of the synthesized NPs and the specific site of interaction of Ce salt on curcumin were evaluated using FTIR spectroscopy. For this experiment, the synthesized CeO_2_ NPs were lyophilized by Telstar freeze dryers (Lyo Quest −85), and the obtained dried powder was blended with KBr and pressed to form a pellet. The hydrodynamic diameter (average NP size), PDI, and zeta potential of as-synthesized NPs were assessed using a Zeta sizer. The morphology, actual size, uniformity, and size distribution of the synthesized NPs were obtained using the TEM imaging technique. The NPs were drop-casted on the TEM grid (a carbon-coated copper grid) and let down to air dry. The imaging was performed at 200 kV accelerating voltage. Semi-quantitative elemental analysis of the synthesized NPs was recorded by EDX analysis. The crystallinity state of as-synthesized NPs was evaluated based on the powder XRD experiments.

### 2.5 Fabrication of the scaffold

In this experiment, a scaffold was fabricated from Alginate (Alg) biopolymer. The Alg powder was dissolved in DI water with the final concentration of 2% w/v and stirred for 1 day to completely dissolve the biopolymer. After completely dissolving the biopolymer, the cross-linker (CaCl_2_, 100 mM) solution was added to the as-prepared polymeric solution. Since Alg is an anionic polymer, cationic substances (e.g., CaCl_2_) can physically cross-link the Alg monomers to form a relatively stable hydrogel network. For the fabrication of NP-bearing scaffolds, first, as-synthesized CeO_2_ NPs were dispersed into the DI water, stirred for 1 day at RT, and then sonicated several times to thoroughly disperse the NPs. Then, the biopolymer (Alg) was added to the dispersion with the final concentration of 2% w/v and stirred for 24h at RT. Finally, the cross-linker (CaCl_2_, 100 mM) was added to the dispersion and gently shacked to form hydrogel-bearing NPs. The fabricated structures were soaked in DI water for 12 h and the DI water was replaced with a fresh one to wash out unreacted substances (Alg and a cross-linker). The resultant samples were lyophilized by Telstar freeze dryers (Lyo Quest −85) and stored for further experiments and applications.

### 2.6 Characterization of scaffolds

The morphology of as-prepared scaffolds was assessed and visualized using the SEM imaging technique. The lyophilized specimens were crashed (to visualize the internal structure) and covered with conducting elements to properly image. The water uptake and retention capacity of the scaffolds were investigated in PBS solution (pH: 7.4) using the weighing method. The specimens were weighed (W_0_), soaked in PBS for 1 day, and weighed again (W_1_). Formulation 1 was utilized to calculate the water uptake capacity.

$$Water\;uptake\left(\%\right)=\left(\frac{W_{1\;-}W_{0}}{W_{0}}\right)\times 100$$ (1)

The porosity of the structure was measured based on the soaking of the scaffolds into a solution (usually ETOH), letting the solution fill the scaffold pores, and calculating the solution volume changes. Briefly, the initial volume of the solution was measured; the scaffolds were soaked in the solution for 1 h and the solution volume was measured, and finally, the scaffold was removed and the solution volume was measured again.

### 2.7 The biological evaluations

#### 2.7.1 The antioxidant activity

The antioxidant potential of the synthesized NPs and NP-bearing scaffolds was assessed using the DPPH assay kit. This experiment is based on the ability of substances/structures (the synthesized NPs and NP-bearing scaffolds in this study) to scavenge and neutralize the free radical (1, 1-diphenyl-2-picril-hydrazine) which is a purple-colored substance that turns yellow after scavenging. The samples were incubated with the reagent (1 mL of DPPH 0.1 mM in methanol), and the absorbance of the solution was read at 517 nm using a microplate reader.

#### 2.7.2 Cell toxicity assay

The viability of the fibroblast L929 cells under incubation with as-prepared structures was measured and assessed using the MTT assay protocol. The cells were grown until 70% confluence in a cell culture medium (DMEL) containing FBS (10%), and Pen/Strep (1%) at 37°C under 5% CO_2_ in a cell culture incubator. Then, the cells were trypsinized, collected from the cell culture flask, re-dispersed in cell culture medium, and incubated with sterilized scaffolds (with ETOH 70%) in a density of 1×10^4^ cells/scaffold. The wells were incubated in a standard cell culture condition (at 37°C under 5% CO_2_ in a cell culture incubator) for 1 and 3 days. At each time point, the cell culture medium was carefully removed and the cells or scaffolds were incubated with MTT salt (0.5 mg/mL) for 4 h at 37°C in a dark environment. Subsequently, the appropriate solvent (DMSO) was added to the system, and the absorbance of the solution was measured at 570 nm using a microplate reader.

#### 2.7.3 Animal experiments

**FIGURE 1:**
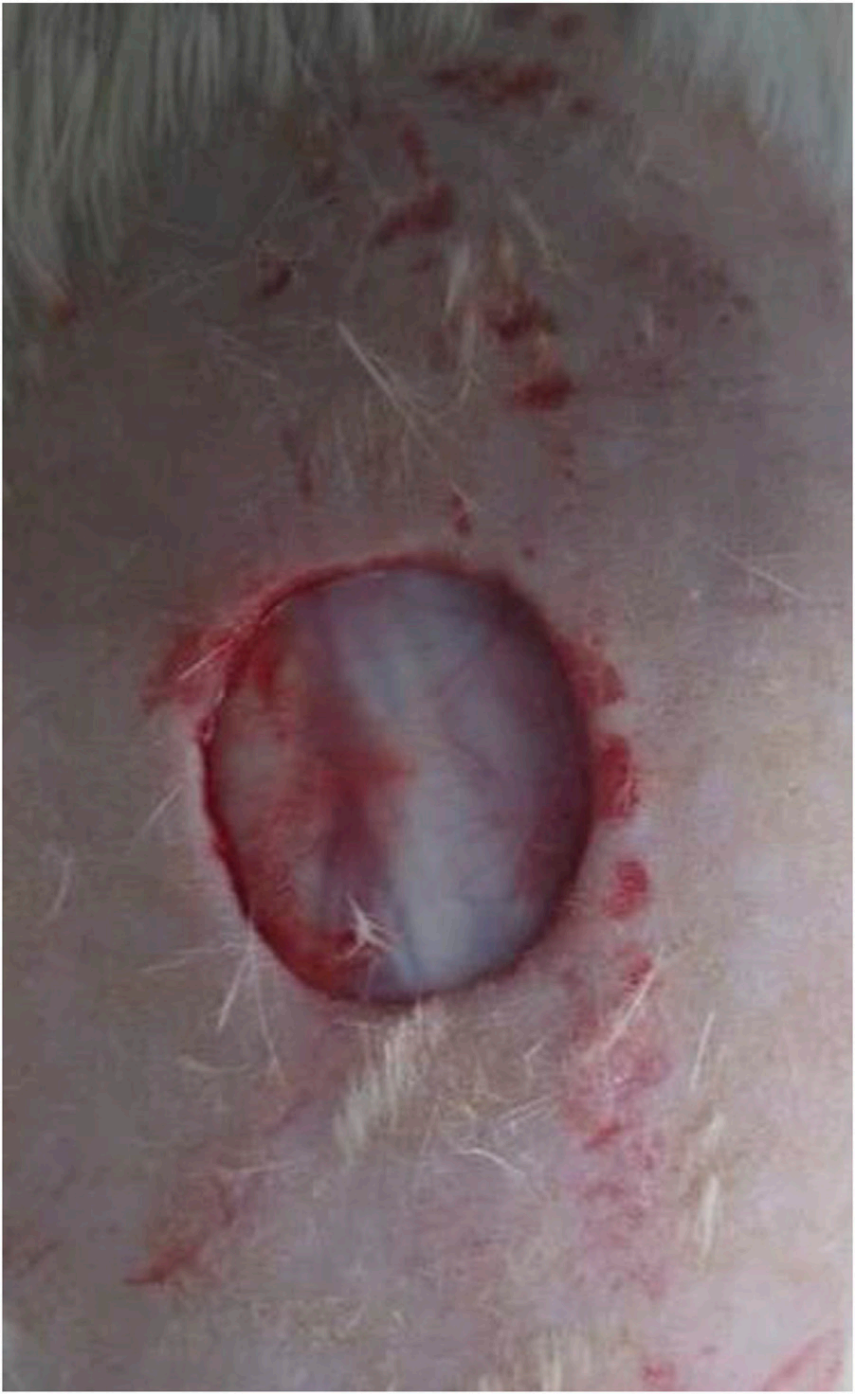
Induced wound on the back of a rat. The full-thickness wounds (1 cm in diameter) were induced in the dorsum via a 10 mm sterile disposable biopsy punch.

The *in vivo* studies were conducted on Male Sprague-Dawley rats based on the approved guidelines. The animals with weights of 240 ± 20 g were housed in separate cages individually at 25°C with proper light/dark cycle (12:12) and free access to water and food. The skin’s full thickness was induced based on previous studies with a slight modification. The rats were anesthetized through the IP injection of ketamine/xylazine (1:1), and the dorsum was shaved and cleaned with povidone-iodine and 70% ethanol. The full-thickness wounds (1 cm in diameter) were induced in the dorsum via a 10 mm sterile disposable biopsy punch (Figure 1). The induced wounds were treated by the fabricated biomaterials. The histological evaluations were conducted 14 days post-treatment to observe and evaluate the efficacy of the applied treatment. For this experiment, a 10 mm biopsy punch was applied to harvest the biopsies. The samples were soaked in a 10% neutral buffered formalin for fixation for 1 day. Then, the samples were subjected to tissue processing, and the slices were stained with Hematoxylin and Eosin (H&E) staining.

## 3 Results and discussion

### 3.1 Characteristics of CeO_2_ NPs

In this project, we utilized a green synthesis approach to produce biogenic CeO_2_ NPs. In this case, a natural substance (curcumin in our study) served as both the reducing and capping/stabilizing agent. Additionally, the resulting NPs obtained from this approach inherit certain biological activities imparted by curcumin. Curcumin is widely recognized for its positive biological properties including antioxidant, anti-inflammatory, antimicrobial, and antitumoral activities. Some of these activities are suitable for regenerative medicine and wound-healing applications. The Indian spice turmeric (Curcuma longa) has been used for millennia in herbal therapy to treat conditions as diverse as rheumatism, diabetic ulcers, anorexia, cough, and sinusitis. The yellow pigment in turmeric comes from a compound called curcumin (diferuloylmethane). Anti-inflammatory, anti-oxidant, anti-carcinogenic, anti-mutagenic, anti-coagulant, and anti-infectious properties have all been seen in curcumin. The anti-inflammatory and antioxidant effects of curcumin are complemented by its demonstrated effectiveness in treating wounds. It speeds up recovery by influencing multiple points in the body’s innate wound-healing process. We applied curcumin as the reducing agent and capping agent in the synthesis process of CeO_2_NPs and characterized the NPs with proper and informative methods.

**FIGURE 2:**
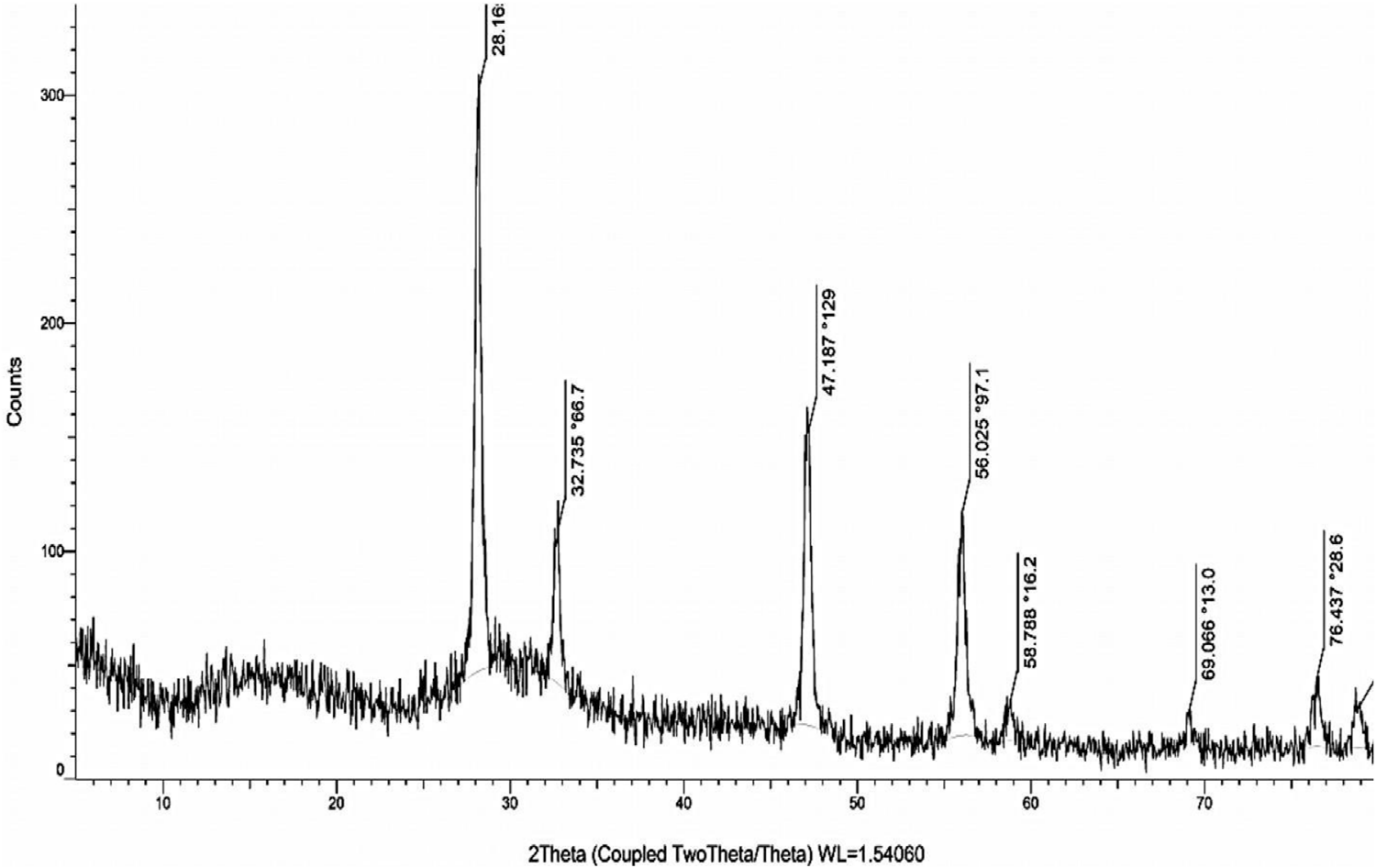
XRD pattern of the Curcumin assisted biosynthesized CeO_2_NPs. XRD pattern is well matched to the XRD pattern of the standard sample (card no 96–900-9009) according to the Joint Committee on Powder Diffraction Standards (JCPDS).

The crystallinity of nanomaterials has a significant role in the performance of the nanomaterials in the intended application. The results of the XRD analysis (Figure 2) showed that the obtained XRD pattern is well matched to the XRD pattern of the standard sample (card no 96-900-9009) according to the Joint Committee on Powder Diffraction Standards (JCPDS). The peaks located at 28.1, 32.7, 47.1, 56.0, 58.7, 69.0, and 76.4 corresponded to (111), (200), (220), (311), (222), (400), and (331) crystallographic planes. There were no additional peaks in the obtained XRD pattern that indicate the purity of bioproduced NPs and efficacy of the applied green synthesis. According to the results and obtained crystal lattice, the biosynthesized NPs had a single-phase cubic fluorite structure with face-centered cubic fashion (a = b = c = 5.14, *α* = β = γ = 90°) and each cerium site was occupied by eight oxygen sites. We applied the Debye–Scherrer equation and observed that the approximate crystallite size of the biosynthesized NPs was around 8.2 nm based on the most intensive broad Bragg peak at 28.1°. Previous studies also reported the possibility of the synthesis of sophisticated CeONPs based on green chemistry methods. Maqbool et al. used *Olea europaea* leaf extract to synthesize CeO_2_NPs and observed that the resultant NPs possessed a single-face cubic center (fluorite structure) (Maqbool et al., 2016).

**FIGURE 3:**
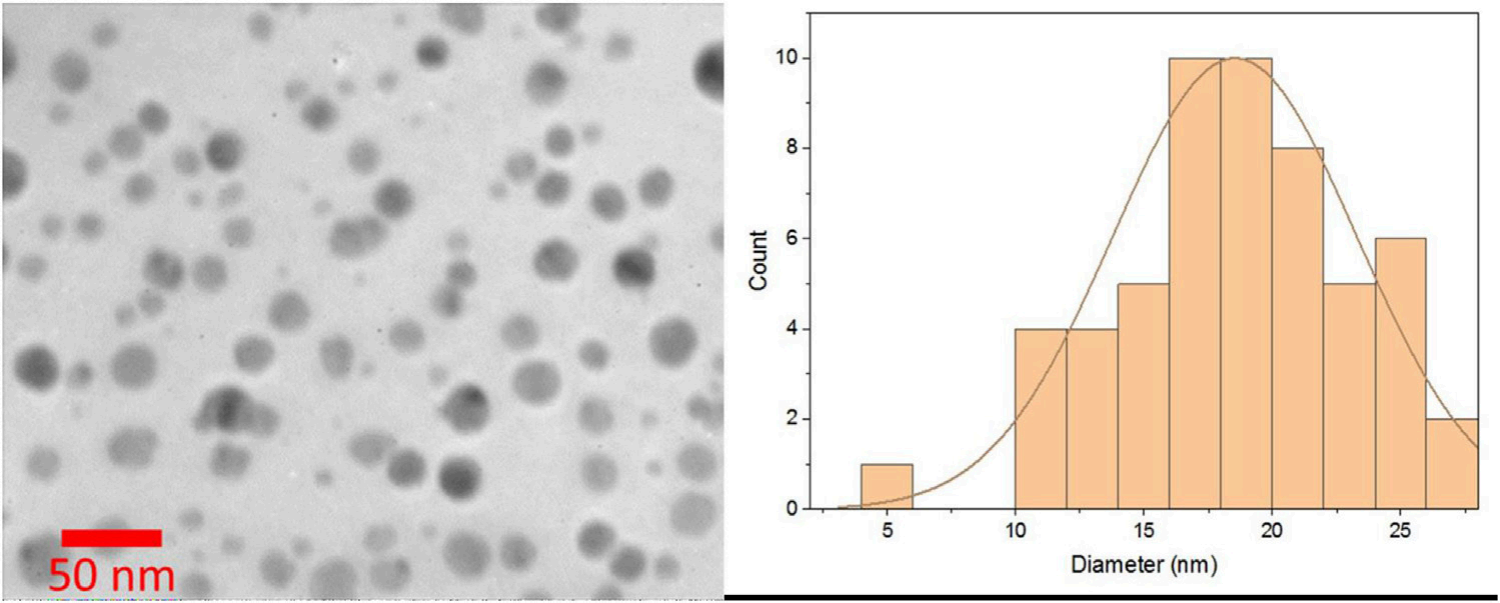
TEM micrograph of curcumin-assisted biosynthesized CeO_2_NPs. The results showed that the NPs are relatively spherical and are well dispersed.

We applied the TEM technique to observe the morphology and measure the actual size of the curcumin-assisted biosynthesized CeO_2_NPs. TEM is a powerful and sophisticated imaging technique for visualizing objects with a size lower than 100 nm. The results (Figure 3) showed that the resultant NPs are relatively spherical in shape and are well dispersed. The image analysis on the obtained TEM images showed that the curcumin-assisted biosynthesized CeO_2_NPs have a size of 18.8 ± 4.1 nm. Maqbool et al. (2016). Synthesized CeO_2_NPs from *O. europaea* leaf extract and reported that the green-produced CeO_2_NPs had highly homogenous symmetry, a spherical shape, and an average particle size of 24 nm.

**FIGURE 4:**
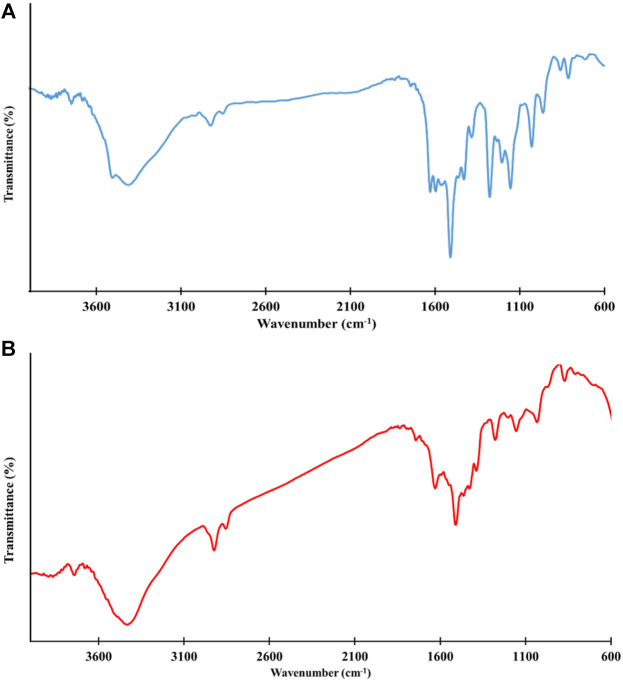
FTIR spectrum of pure curcumin **(A)** and curcumin-assisted biosynthesized CeO2NPs **(B)**.

The FTIR spectroscopy was applied to identify the surface functional groups of pure curcumin and curcumin-assisted biosynthesized CeO_2_NPs and the results are presented in Figure 4. The intense band located at around 3,500 cm^–1^ relates to the stretching vibration of hydrogen-bonded O–H of Curcumin. The bond around 2,920 and 2,850 cm^–1^ can be attributed to asymmetric stretching vibrations of Csp2–H and Csp3–H bonds, respectively. The peak centered at around 1,627 cm^–1^ is related to the C–H frequency of the aromatic overtone. The peaks at around 1,450 and 1,420 cm^–1^ are related to the aromatic stretching vibrations of the benzene ring. The sharp peak at 1,510 cm^–1^ originates from the stretching vibration of the conjugated carbonyl (C=O) (Pawar et al., 2018; Hettiarachchi et al., 2021; Chen et al., 2023).

### 3.2 Characteristics of hydrogel nanocomposite

**FIGURE 5:**
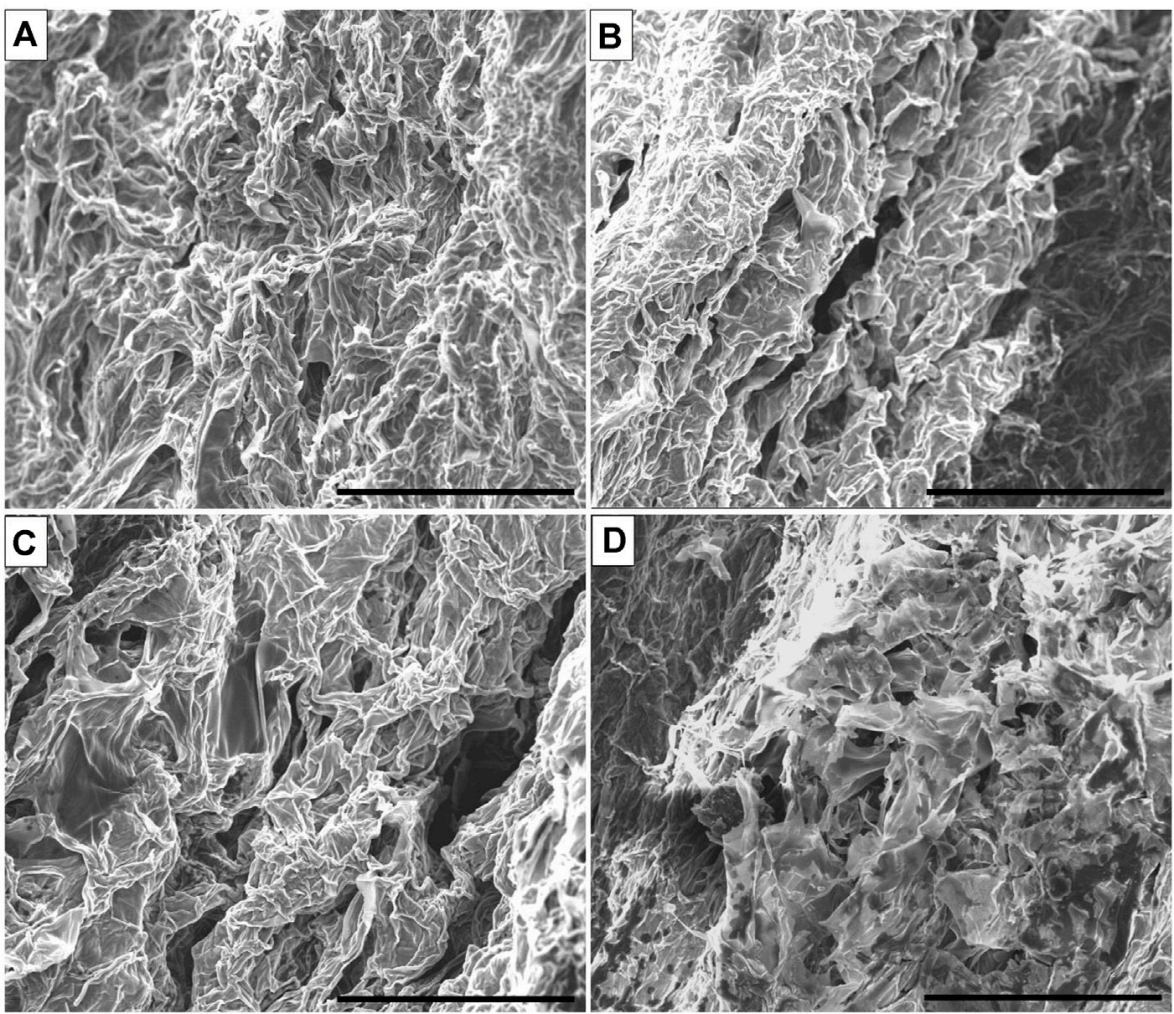
SEM images of the fabricated pure Alg hydrogel **(A)**, Alg-CeO_2_ 3% hydrogel **(B)**, Alg-CeO_2_ 5% hydrogel **(C)**, and Alg-CeO_2_ 7% hydrogel **(D)**. Scale bar: 300 μm.

Hydrogel dressings, comprising complex hydrophilic polymers, exhibit a high water content, typically up to 90%. As the name suggests, hydrogels assist in maintaining hydration, facilitating rehydration, and promoting autolytic debridement of wounds. Examples of hydrogels include sheet dressings, amorphous gels, and dressings impregnated with hydrogel sheets—all composed of water-swelling, insoluble polymers. Hydrogels foster a moist environment conducive to cell migration and possess the capacity to absorb certain wound exudates. Notably, hydrogel dressings offer the additional advantage of autolytic debridement without disturbing granulation or epithelial cells. These hydrogels are effective in the breakdown of slough on the wound surface and are suitable for wounds ranging from dry to mildly exuding (Weller and Team, 2019; Demeter et al., 2023; Jia et al., 2023; Qiao et al., 2023). Based on the aforementioned qualities, hydrogels are a promising product for use as wound dressings. In addition to offering physical protection and keeping the microenvironment moist, the antioxidative, antibacterial, and injectable hydrogel wound dressings now under development can also aid the healing process by influencing the phase of wound repair. Antioxidant hydrogels, for instance, have been shown to remove excess reactive oxygen species in chronic wounds, thereby reducing oxidative stress, enhancing the wound microenvironment, supporting collagen production and re-epithelialization, and decreasing the wound’s pH value to speed up healing and reduce infection (Lei et al., 2020). Figure 5 represents SEM images of the fabricated hydrogels.

**FIGURE 6:**
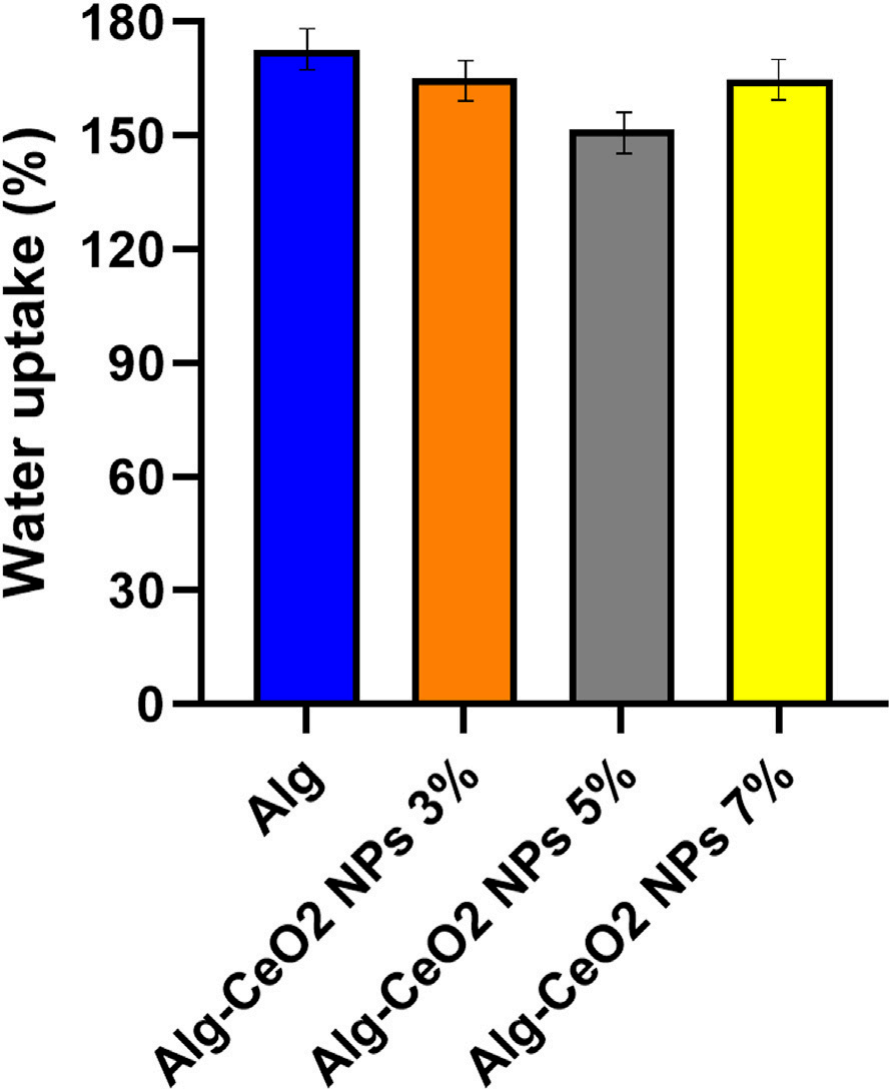
Water uptake capacity of the fabricated hydrogels. To promote the synthesis of a new extracellular matrix (ECM), an ideal skin scaffold should possess the capability to absorb water at a range of 100–800 times its dry weight without undergoing swelling.

By utilizing hydrogel-based wound dressings, a favorable moist environment can be established, aiding the healing process. This approach of moist wound healing contrasts with the traditional medical approach of forming a scab. Interestingly, epithelialization occurs twice as quickly in humid occlusive/semi-occlusive environments compared to dry ones. While advanced wound care dressings effectively maintain moisture and accelerate healing, excessive moisture within the wound bed may lead to maceration and tissue deterioration. The optimum skin scaffold or wound dressing, as indicated above, should avoid drying the wound in addition to maintaining a moist environment and be able to drain the excessive wound exudate. To facilitate the production of new ECM, a good skin scaffold should be able to absorb between 100 and 800 times its dry weight in water without becoming swollen. Excessive pain is experienced by the patient due to the accumulation of wound exudate at the wounded site, which degrades ECM components and involves the surrounding tissues (Morgado et al., 2015; Nosrati et al., 2021; Wang et al., 2023; Lu et al., 2022). Figure 6 shows the water uptake capacity of the hydrogels.

**FIGURE 7:**
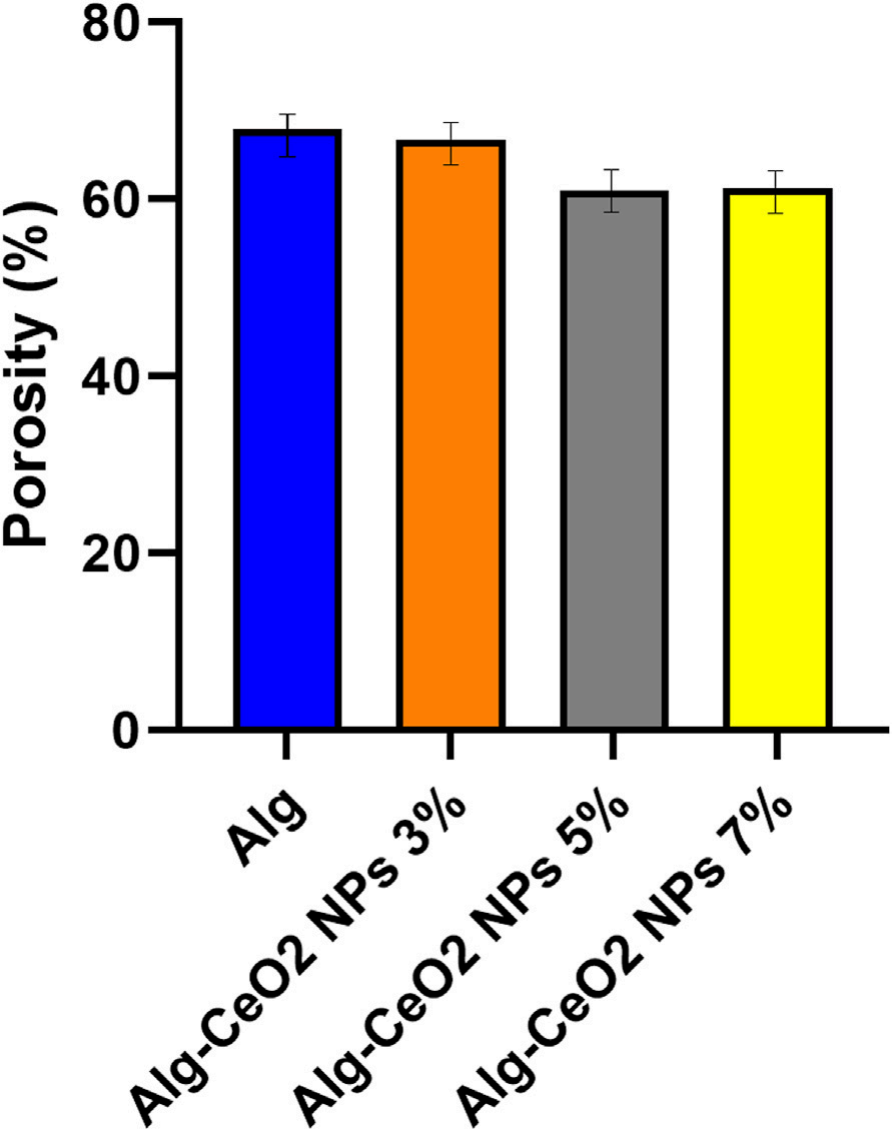
Porosity percentage of the fabricated hydrogels. The porosity of wound dressings and healing structures plays a pivotal role in skin tissue engineering. It is critical as it creates essential gaps for cell inhabitation, proliferation, migration, and differentiation.

Figure 7 shows the porosity of the fabricated hydrogels. The porosity of a wound dressing/healing structure is crucial in skin tissue engineering because it provides the necessary gaps for cell habitation, proliferation, migration, and differentiation. Furthermore, the porous 3D matrix of the scaffold aids in supplying oxygen and nutrients to the injured skin, reducing the risk of infection. Structures with a porosity of 60%–90% have been found to be effective for wound healing, as they allow for adequate cellular function, oxygen and nutrient transport, and the synthesis of new ECM. The scaffold’s porosity and mechanical qualities should be in harmony because increasing the scaffold’s porosity decreases the aforementioned mechanical parameters. Nanobiomaterials, such as carbon nanotubes and ceramic or metallic nanoparticles, can be employed to prevent a major decrease in the scaffold’s mechanical qualities and, in addition, to encourage angiogenesis (Freyman et al., 2001; Boccaccini et al., 2010; Karuppuswamy et al., 2014; Nosrati et al., 2021).

### 3.3 Bioevaluation results

**FIGURE 8:**
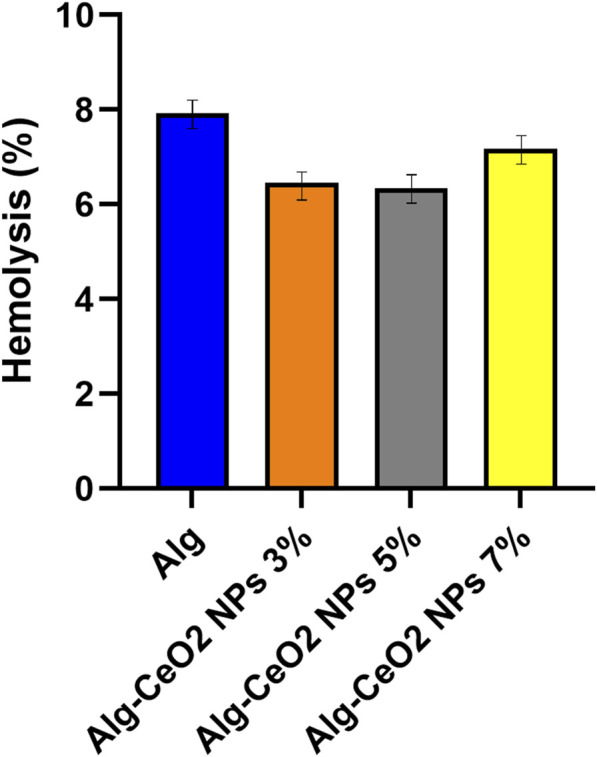
Hemolysis induced by the fabricated hydrogels. The treatment with the fabricated structures demonstrated Hb leakage values below 10%, indicating minimal hemolysis induction by the scaffolds.

Patient safety is the primary concern while designing medical dressings. It is well known at this point that hemolysis can affect the wound-healing process. Vascularization, for instance, requires active nitric oxide (NO), and the free hemoglobin (Hb) released from damaged RBCs can trigger NO inactivation, which in turn slows down the healing process. Fibroblast growth and collagen remodeling are also negatively affected by free Hb (Herold et al., 2001; Jensen, 2009; Bernatchez et al., 2013; Qu et al., 2018; Zare-Gachi et al., 2020). The results of the Hb leakage under treatment with the fabricated structures (Figure 8) showed that the scaffolds induced hemolysis lower than 10%. It can be concluded that the obtained scaffolds are hemocompatible.

**FIGURE 9:**
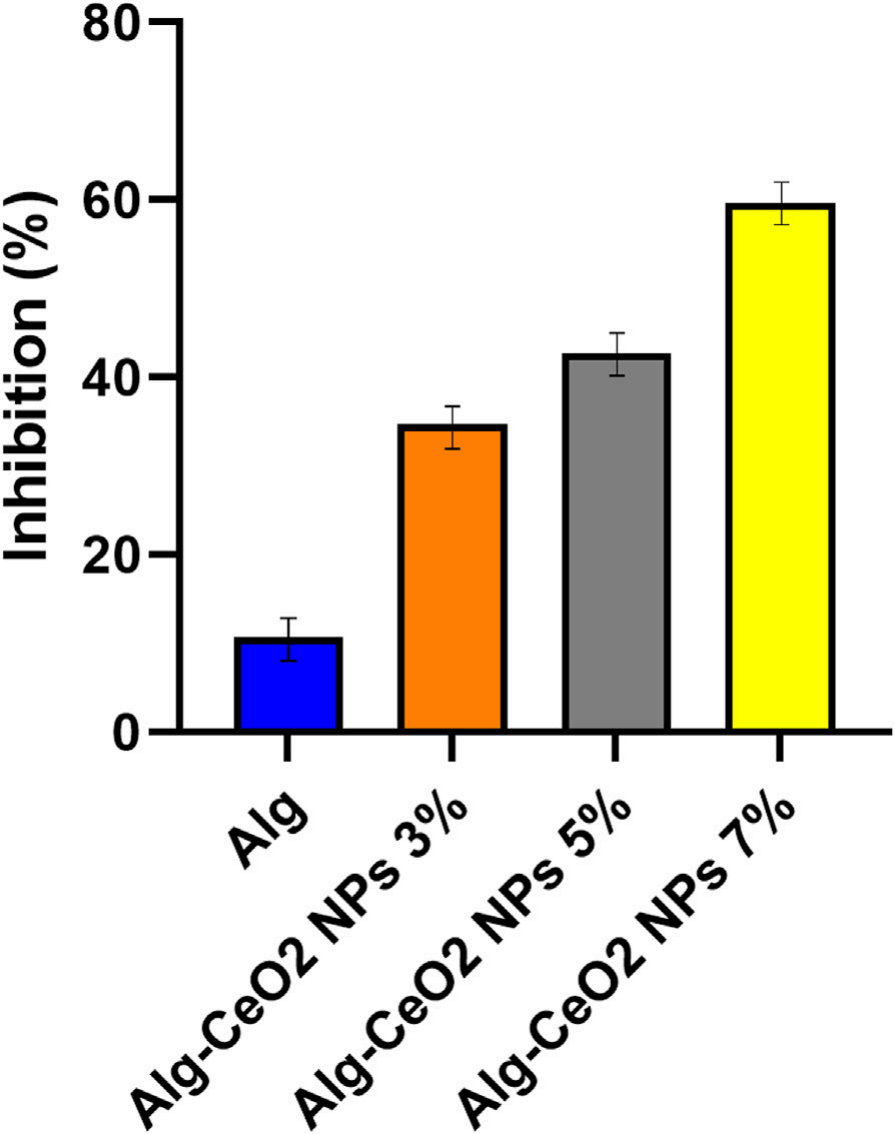
Inhibitory effect of the hydrogels on DPPH radical. The evaluation of radical scavenging potential revealed that the synthesized CeO2 nanoparticles exhibit remarkable antioxidant activity. The results suggest that these nanoparticles possess strong capabilities in scavenging radicals.

An imbalance between the body’s pro- and antioxidant defenses can lead to oxidative stress, which has significant implications. During this process, the production of ROS (Reactive Oxygen Species) increases, playing a crucial role in wound healing. ROS, in low levels, regulates multiple signal transduction pathways within cells and provides energy to phagocytes for bacteria phagocytosis. However, excessive active oxygen can have predominantly negative effects on biological systems. High concentrations of ROS directly interact with lipids, proteins, and DNA in cells, causing damage and even cell death (Guo and DiPietro, 2010; Jiang et al., 2011; Dunnill et al., 2017; Cheng et al., 2023; Park et al., 2023). CeO_2_ has been thoroughly explored for its controlled synthesis and catalytic uses due to its plentiful oxygen vacancies and reversible transition between Ce (iii) and Ce (iv) 3. The ability of CeO_2_ NPs to neutralize abundant ROS and RNS has recently come to light, sparking interest in their potential biological applications. CeO_2_ NPs can protect a wide range of oxidative stress-related ailments, including chronic inflammation, ischemic stroke, and neurological disorders, according to preliminary biological investigations. CeO_2_ NPs in particular have been shown to hasten the healing of cutaneous wounds by promoting the proliferation and migration of key skin-generating cells (Sun et al., 2012; Montini et al., 2016). The results of the radical scavenging potential evaluation showed that of the synthesized CeO_2_, NPs have remarkable antioxidant activity (Figure 9). Moreover, the incorporation of the synthesized CeO_2_ NPs into the Alg hydrogel endowed the antioxidant potential to the structure.

**FIGURE 10:**
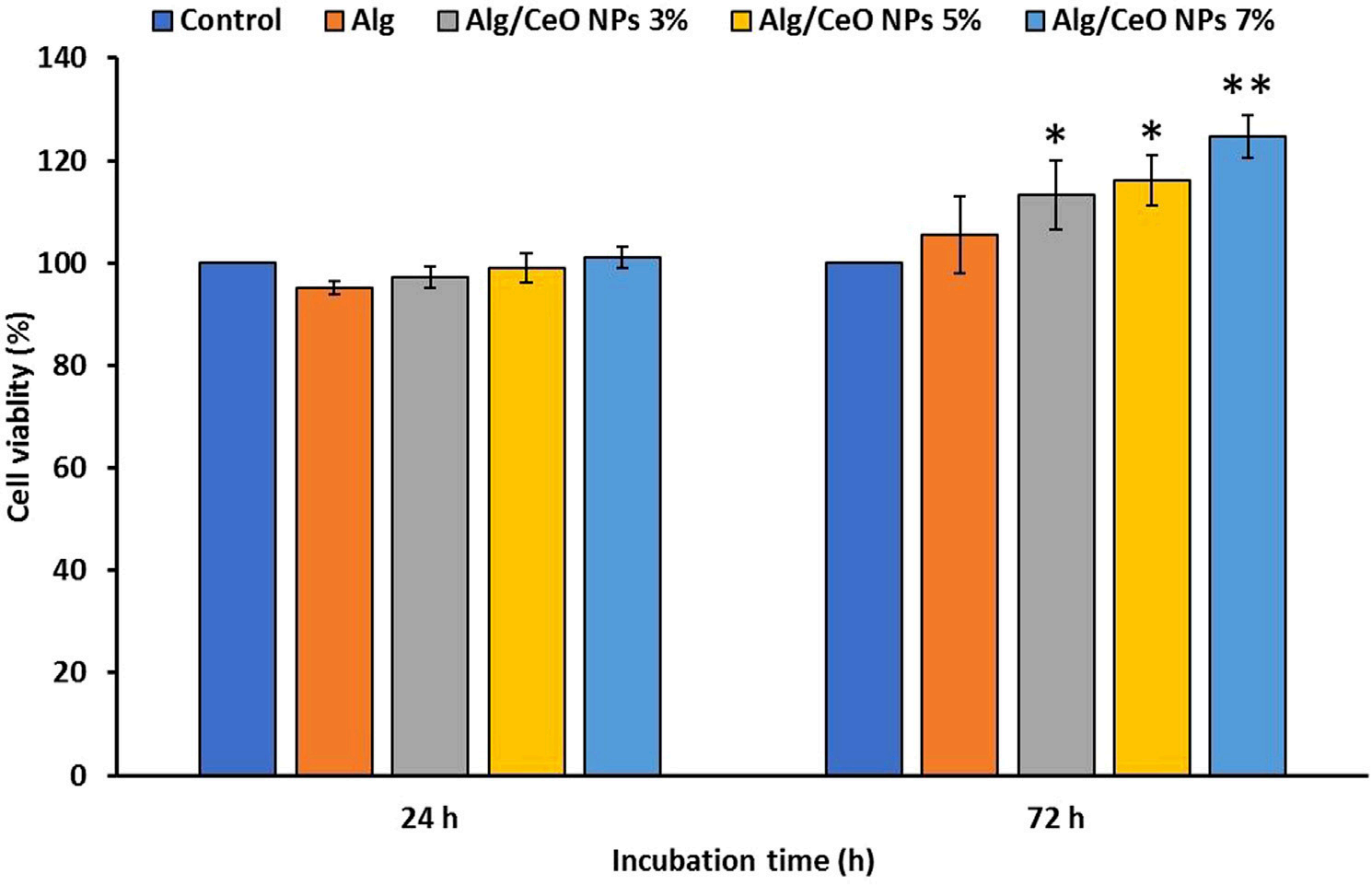
Effect of the hydrogels on the viability of fibroblast cells evaluated by the MTT assay. Developed wound-healing hydrogels exhibited both biocompatibility and a stimulatory effect on cell proliferation. The synthesized hydrogels not only supported cell growth but also contributed to the healing process. Data represented as Mean ± SD, *: *p* < 0.05, **: *p* < 0.001.

MTT assay was conducted to assess the cytotoxicity of the samples and their effects on the viability and proliferation of cells (Figure 10). The results showed that the fabricated wound healing hydrogels not only were biocompatible but also induced proliferative effects on the cells. The beneficial effects of CeO_2_ NPs on wound healing-involved cells (e.g., keratinocytes, fibroblasts, and vascular endothelial cells) have been reported by other researchers (Chigurupati et al., 2013).

**FIGURE 11:**
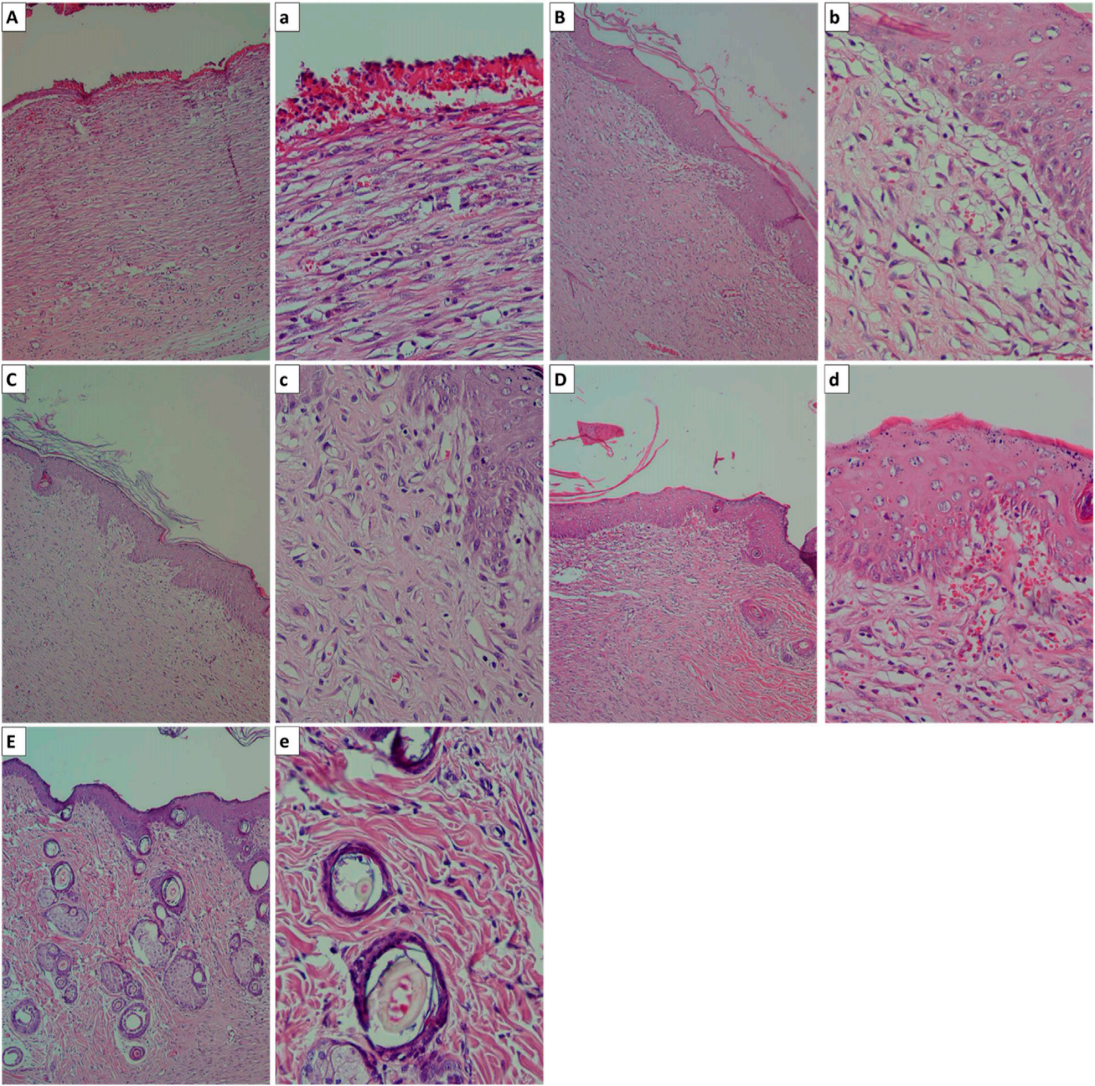
The histopathological observation of the wounds. **(A)** and **(a):** negative control (wound without treatment, **(B)** and **(b):** wound treated with pure alginate hydrogel, **(C)** and **(c):** wound treated with Alg/CeO NPs 3%, **(D)** and **(d):** wound treated with Alg/CeO NPs 5%, and **(E)** and **(e):** wound treated with Alg/CeO NPs 7%.

The process of wound healing is a dynamic and complex one that ultimately results in the injured tissue regaining its normal anatomy and functioning normally. Yet, unusually prolonged inflammation produces the production of excessive cytotoxic enzymes, inflammatory mediators, free radicals, and cytokines, all of which cause significant cell damage to the surrounding tissue and pave the way for the proliferative phase. The excessive creation of free radicals also produces oxidative stress, which leads to cytotoxic effects that are harmful and cause delays in the healing process of wounds. As a result, the utilization of a substance that is both anti-inflammatory and anti-oxidative in order to mitigate chronic inflammation and cut down on an excessive amount of free radicals might be an essential method for improving wound healing (Dissemond et al., 2002; Kant et al., 2014; Huang et al., 2018). The beneficial effects of CeO_2_ NPs and CeO_2_ NPs-embedded structures on the wound healing process have been reported by other studies. Chigurupati et al. reported that water-soluble CeO_2_ NPs accelerated the healing of full-thickness dermal wounds in mice. They proposed that enhancement of the migration and proliferation and migration of fibroblasts, keratinocytes, and vascular endothelial cells under treatment with water-soluble CeO_2_ NPs are involved mechanisms (Chigurupati et al., 2013). Huang et al. fabricated chitosan-coated CeO_2_ nanocubes and applied them as wound-healing biomaterials in excision wounds on adult Sprague Dawley rats. They observed that the wound-healing potential of the applied structure was higher than the recombinant human epidermal growth factor (rhEGF). They proposed that the observed remarkable wound-healing potential can be due to the antioxidant potential of CeO_2_ nanocubes since they reduced the expression of tumor necrosis factor-alpha (an inflammatory cytokine) and increased the expression of interleukin-10 (an anti-inflammatory cytokine) (Huang et al., 2018). The histopathological evaluations (Figure 11) revealed that the treatment of the induced wound with CeO_2_ NP-bearing hydrogels enhanced the wound-healing process.

## 4 Conclusion

Refractory wounds and ulcers are a major contributor to the quality of life decline seen in people with oxidative stress-related disorders like diabetes. Hyperglycemia-induced oxidative stress in refractory diabetic ulcers disrupts vascular endothelial cells and worsens inflammation, while also activating a number of aberrant metabolic pathways that collectively limit wound healing. In the current experiment, we synthesized CeO NPs via a green chemistry approach and used curcumin as the reducing and capping agent. The synthesized NPs exhibited the desired properties. The NPs were incorporated into Alg hydrogel and applied as wound-healing biomaterials. As a result, we proposed that CeO NP-bearing Alg hydrogels with significant anti-inflammatory and anti-oxidative capabilities could be employed to treat these recalcitrant wounds caused by oxidative stress. Moreover, for the future direction of this study, the fabricated CeO NP-bearing Alg hydrogels can be applied for diabetic wound healing. The non-healing of chronic diabetes wounds can be attributed to several key factors, including inadequate cell proliferation, impaired cell migration, and insufficient angiogenesis. The utilization of cerium oxide nanoparticles (nCeO2) in the composition of wound dressings holds significant potential as a viable strategy for enhancing angiogenesis and facilitating the healing process of diabetic wounds. CeO_2_NPs possess properties that make them well-suited for use as diabetic wound-healing materials due to their antioxidant, anti-inflammatory, and regenerative attributes. Research findings indicate that CeO_2_NPs have the potential to promote wound healing, facilitate tissue regeneration, and mitigate scar formation. Cerium oxide nanoparticles (CeO_2_NPs) have the potential to mitigate bacterial infections and enhance immune responses at wound sites.

## Data Availability

The datasets presented in this study can be found in online repositories. The names of the repository/repositories and accession number(s) can be found in the article/Supplementary Material.
