# Corrigendum: *In vitro* and *in vivo* evaluation of alginate hydrogel-based wound dressing loaded with green chemistry cerium oxide nanoparticles

**DOI:** 10.3389/fchem.2024.1373205

**Published:** 2024-02-01

**Authors:** Ran Zhao, Chenyuyao Zhao, Yi Wan, Muhammad Majid, Syed Qamar Abbas, Yibing Wang

**Affiliations:** ^1^ Burn and Plastic Surgery, Shandong Provincial Hospital Affiliated to Shandong First Medical University, Jinan, Shandong, China; ^2^ Key Laboratory of Biopharmaceuticals, Postdoctoral Scientific Research Workstation, Shandong Academy of Pharmaceutical Science, Jinan, Shandong, China; ^3^ Graduate School, Shandong First Medical University, Jinan, Shandong, China; ^4^ School of Mechanical Engineering, Shandong University, Jinan, Shandong, China; ^5^ Faculty of Pharmacy, Hamdard University, Islamabad, Pakistan; ^6^ Department of Pharmacy, Sarhad University of Science and Technology, Peshawar, Pakistan

**Keywords:** wound healing, alginate, cerium oxide nanoparticles, curcumin, hydrogel

In the published article, there was an error in Figure 11 as published. The incorrect version of the figure was mistakenly uploaded due to an oversight. The corrected Figure 11 and its caption appear below.

**FIGURE 11:**
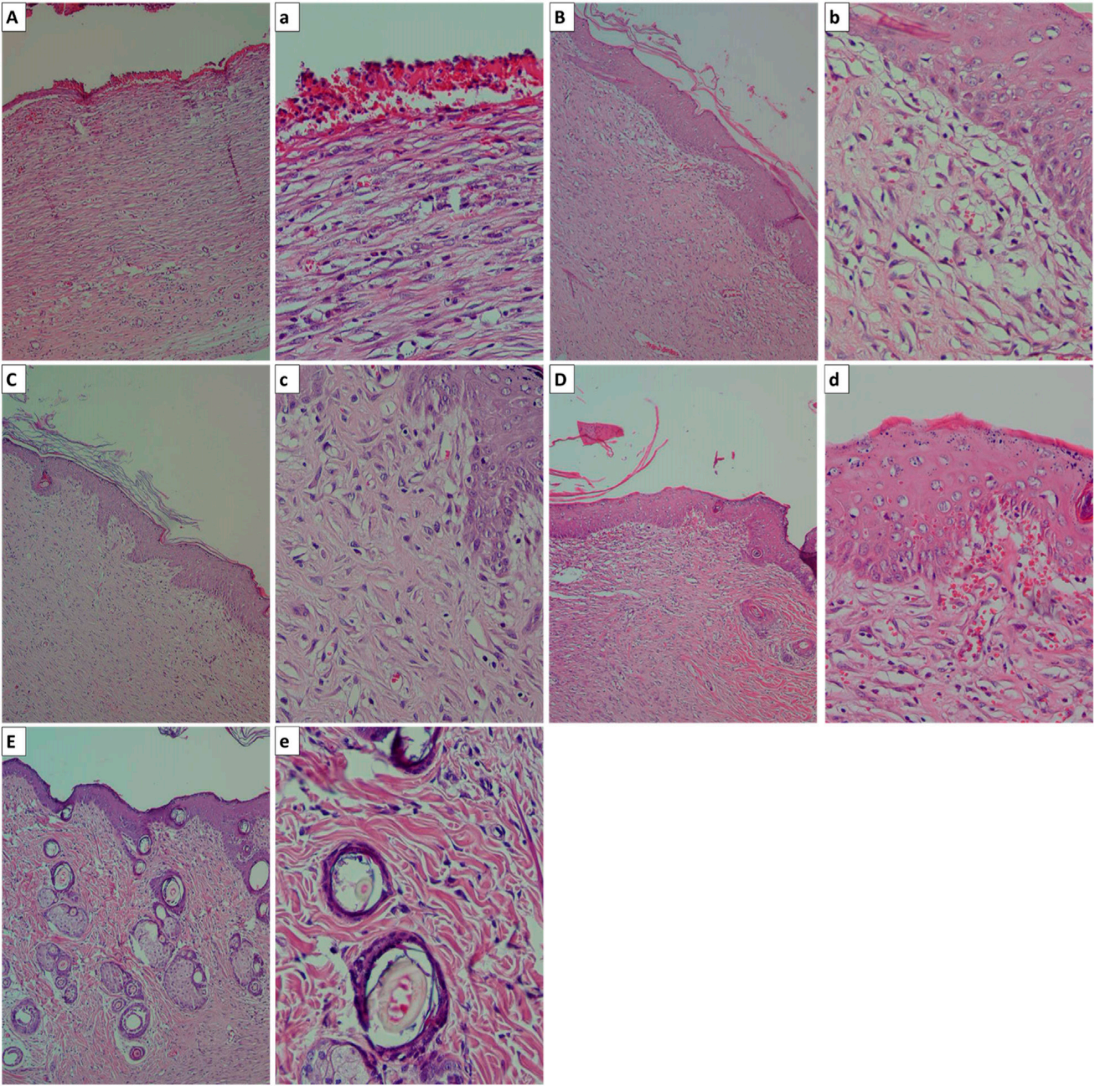
The histopathological observation of the wounds. **(A)** and **(a)**: negative control (wound without treatment, **(B)** and **(b)**: wound treated with pure alginate hydrogel, **(C)** and **(c)**: wound treated with Alg/CeO NPs 3%, **(D)** and **(d)**: wound treated with Alg/CeO NPs 5%, and **(E)** and **(e)**: wound treated with Alg/CeO NPs 7%.

The authors apologize for this error and state that this does not change the scientific conclusions of the article in any way. The original article has been updated.

